# Analysing government health expenditure in Africa: Trends, determinants and policy options

**DOI:** 10.4102/jphia.v17i1.1643

**Published:** 2026-05-06

**Authors:** Kelechi J. Uzor, Obinna A. Oty, Chizaram K. Uzor, Uchenna A. Amaechi

**Affiliations:** 1 Harvard Kennedy School, Harvard University, Cambridge, United States of America; 2 Faculty of Primary Care, East Kent Hospital University Foundation Trust, Kent, United Kingdom; 3 Faculty of Business Administration, Hull University, Hull, United Kingdom; 4 Faculty of Global Health, University of Geneva, Geneva, Switzerland

**Keywords:** health financing, African Union, Abuja Declaration, government expenditure, budget, health systems

## Abstract

**Background:**

In April 2001, African Union (AU) Heads of State adopted the Abuja Declaration, pledging to allocate at least 15% of government expenditure to health. More than two decades later, many African health systems remain underfunded, with external aid increasingly unsustainable as official development assistance declines.

**Aim:**

This article examines progress towards the Abuja target, the role of political economy in shaping government health expenditure (GHE), and policy options for sustainable domestic financing.

**Setting:**

The authors examined the Global Health Expenditure across 53 AU Member States.

**Methods:**

The authors analysed the Global Health Expenditure Database on GHE as a proportion of total government expenditure between 2000 and 2022. Additional political economy analysis explored the governance and economic correlates of GHE.

**Results:**

Mean GHE in Africa has stalled, rising only 0.5% points since 2001. In 2022, only South Africa met the 15% Abuja benchmark, with seven countries allocating 10% – 14.9% and 45 countries allocating less than 10%. Compared to slightly better off countries, low-income countries such as Rwanda and Mozambique allocated a larger proportion of their government expenditure to health, indicating the importance of political will.

**Conclusion:**

Progress toward the Abuja target has been slow and uneven, highlighting the need for a multi-pronged approach to expand fiscal space for health, anchored in strong regional governance and accountability frameworks. Strengthening these foundations will be critical to building resilient health systems, reducing reliance on external aid, and accelerating progress toward universal health coverage in Africa.

**Contribution:**

This study advances existing research by demonstrating that political commitment and institutional factors, rather than gross domestic product per capita alone, are key determinants of health spending priorities, thereby challenging the prevailing assumption in the health financing literature that fiscal capacity is the primary driver of health budget prioritisation.

## Introduction

In April 2001, African Union (AU) Heads of State met in Abuja, Nigeria’s capital and committed to an ambitious health financing target. The pledge, known as the Abuja Declaration, stipulated AU Member States to allocate at least 15% of their annual government expenditure to the health sector.^1^ More than two decades following the declaration, many health systems in Africa remain underfunded, leading to poor health outcomes and preventable deaths.

In the years that followed, many African countries, unable to sustainably finance their healthcare systems from domestic resources have become increasingly reliant on official development assistance (ODA) from high-income countries for financial support. Defined by the Organisation for Economic Co-operation and Development (OECD) as ‘government aid that promotes and specifically targets the development and welfare of developing countries’, ODA either as grants or concessional loans make up at least two-thirds of external financing for low-income countries.^2^

In recent times, however, the global donor landscape has undergone significant structural shifts. In the United States, for example, the restructuring of the United States Agency for International Development (USAID) has introduced greater uncertainty into long-term global health appropriations.^3^ These dynamics including the recent America First Global Health Strategy signals a shift towards prioritising national security, supply chain resilience and domestic industrial policy over traditional multilateral health assistance. Similar trends are evident across Europe, where rising inflation, energy insecurity, migration pressures and post-pandemic fiscal tightening have prompted several governments to redirect resources towards domestic economic stabilisation.^4^

As a result, development assistance budgets have come under strain, with global health financing increasingly competing against domestic social spending priorities. Collectively, these shifts signal a structural tightening of concessional financing for health, rather than a temporary fluctuation, fundamentally altering the external financing environment for low- and middle-income countries. This shifting landscape, marked by declining ODA flows to low- and middle-income countries and the persistent inability of many African governments to mobilise sufficient domestic health financing over the past few decades because of slow economic growth, rising debt burdens and competing development priorities emphasises the urgent need for African countries to reimagine their health financing systems to ensure self-reliance and resilience in meeting domestic health priorities.^5^

Given Africa’s vulnerability to infectious disease outbreaks and epidemics, alongside the rising burden of non-communicable diseases driven by demographic transitions and increasing life expectancy, the importance of domestic resource mobilisation in securing the continent’s health security cannot be overstated.^6,7,8^

## Research methods and design

This study analyses World Bank data on domestic general government health expenditure (GHE), which captures government spending on health financed from domestic public sources. The dataset covers 53 AU Member States over the period 2000–2022, spanning the years immediately preceding the Abuja Declaration through the most recent year for which data are available.^9^ With respect to indicator selection, GHE as a percentage of total government expenditure reflects the proportion of public spending allocated to the health sector. While this metric does not measure fiscal effort relative to economic size (unlike health expenditure as a share of gross domestic product [GDP]), it serves as a relevant proxy for government prioritisation of health within national budgets and the extent of domestic public commitment to health financing.

The quantitative analysis employs two complementary statistical approaches. Firstly, the authors computed unweighted regional averages of GHE across AU-defined sub-regions, including Southern, Northern, Central, Eastern and Western Africa. This approach provides a macro-level assessment of regional patterns in public health financing, enabling comparison of average government commitments to health spending across regions and identification of those who have made relatively greater or lesser fiscal effort over time.

Secondly, a descriptive trend analysis of GHE was conducted by examining annual percentage changes in GHE from 2000 to 2022 across the same regions. This approach captures year-to-year dynamics in public health financing, highlighting periods of progress, stagnation or retrenchment at the regional level. Countries with incomplete data in the World Bank database, specifically Somalia (2000–2012) and South Sudan (2000–2016), are excluded from regional averages for the corresponding years to avoid distorting aggregate estimates.

Finally, the quantitative findings are discussed extensively using Heller’s framework, which explores opportunities to expand fiscal space for health,^10^ alongside a review of the quantitative findings through a political economy lens using the Ideas–Interests–Institutions (3Is) framework.^11^ Together, these analytical frameworks were chosen to explore the quantitative findings as they examine how political, economic, and institutional factors shape domestic resource mobilisation for health in selected countries and how they impact the financial sustainability of health programmes.

### Ethical considerations

This article followed all ethical standards for research without direct contact with human or animal subjects. The research relied exclusively on publicly available health financing data.

## Results

**FIGURE 1:**
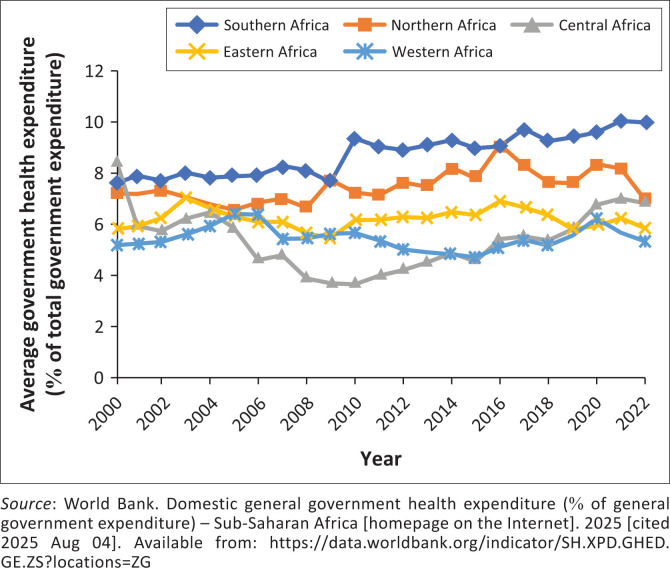
Average government health expenditure as a proportion of total government expenditure across regions in Africa, 2000–2022.

The analyses reveal substantial regional variation in domestic public investment in health across the African continent. As illustrated in Figure 1^9^, the Southern Africa region comprising countries such as South Africa, Botswana, Lesotho, Namibia, Eswatini, Zambia, Mozambique, Angola, Zimbabwe and Malawi exhibited a relatively consistent upward trend in average GHE when examined as a regional average. Regional GHE in Southern Africa increased from approximately 7.6% of total government expenditure in 2000, prior to the Abuja Declaration, to about 10% in 2022.

However, regional averages obscure significant heterogeneity in country-level performance and domestic health financing capacity within the sub-region. In 2022, two upper-middle income countries in Southern Africa, South Africa and Botswana, allocated 16.89% and 14.62% of their total government expenditure to health, respectively, approaching or exceeding the Abuja target. In contrast, Zimbabwe and Malawi, two low-income countries in Southern Africa, allocated only 5.21% and 3.29%, respectively, during the same period.

By comparison, other regions of the continent have experienced greater volatility and, overall, stagnation in domestic allocations to healthcare. In several cases, these regions also recorded a noticeable decline in GHE following the coronavirus disease 2019 (COVID-19) pandemic, reflecting the broader macroeconomic shocks that constrained fiscal space in many low- and middle-income countries.

**FIGURE 2:**
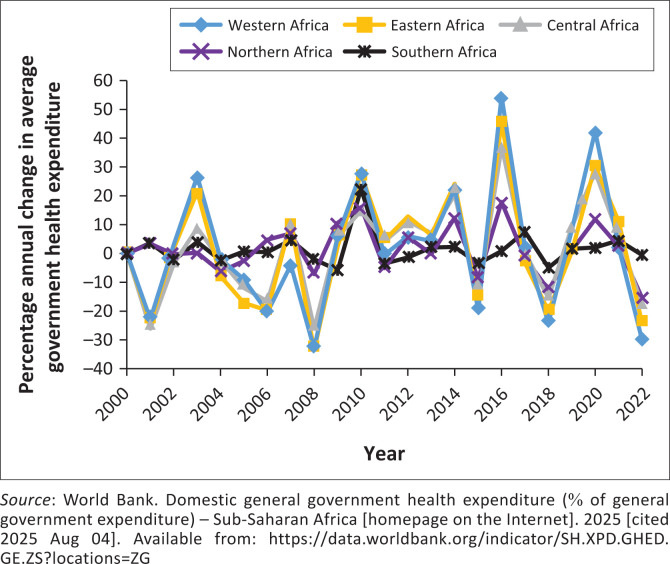
Percentage annual change in average government health expenditure across regions in Africa, 2000–2022.

A descriptive trend analysis of GHE (Figure 2)^9^, examining annual percentage changes from 2000 to 2022 across the same regional groupings, revealed substantial volatility, characterised by pronounced year-to-year fluctuations and notable declines following the onset of the COVID-19 pandemic in 2019. These trends coincided with broader fiscal and macroeconomic pressures across the continent. Among the regions, Western Africa exhibited the greatest year-to-year budgetary variation, while Southern Africa demonstrated comparatively greater stability in GHE allocations over time.

**FIGURE 3:**
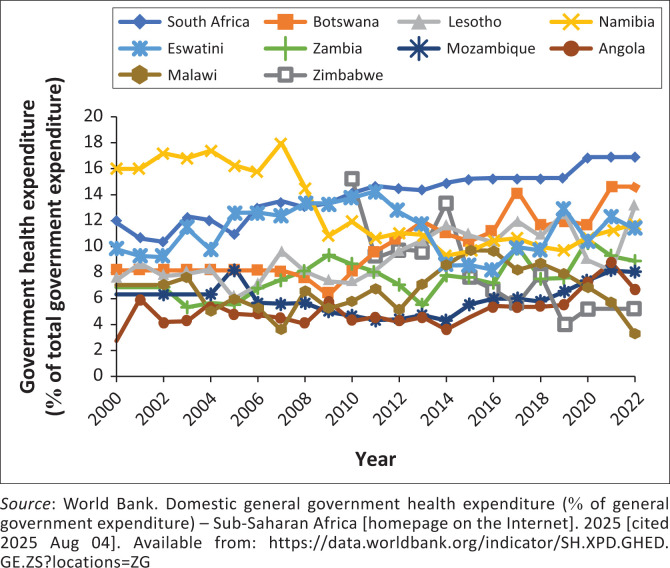
Government health expenditure as a proportion of total government expenditure in Southern Africa, 2000–2022.

A deep-dive analysis of country-level data (Figure 3 to Figure 7)^9^ showed that by 2022, only South Africa met the 15% GHE benchmark stipulated in the Abuja Declaration. While economic advancement, commonly measured by GDP per capita, appears to have contributed to stronger health financing capacity in upper-middle income countries such as South Africa, Botswana and Namibia, patterns of budgetary prioritisation across countries remain highly heterogeneous. For instance, Mozambique, a country in Southern Africa with a GDP per capita of $578.00 in 2022 allocated 8.04% of its total government expenditure to healthcare in the same year.^12^ In that sense, the government allocation for health as a proportion of the total government budget is substantially higher than that of Senegal, which allocated only 3.37% of its government budget to health in 2022, despite having a considerably higher GDP per capita of approximately $1574.00.

**FIGURE 4:**
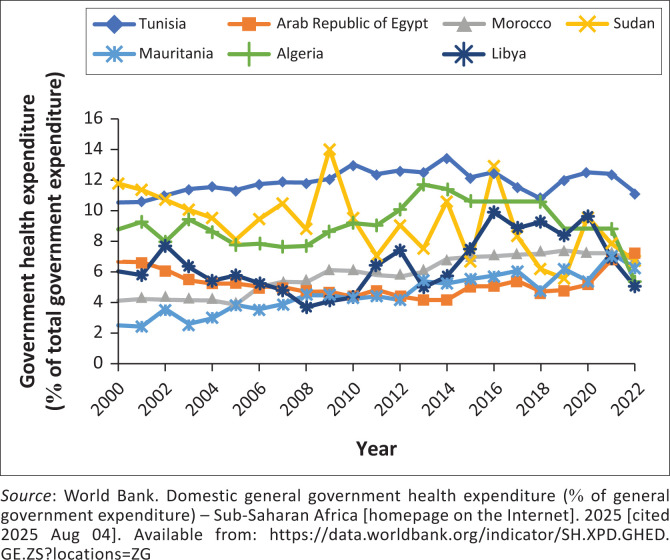
Government health expenditure as a proportion of total government expenditure in Northern Africa, 2000-2022.

**FIGURE 5:**
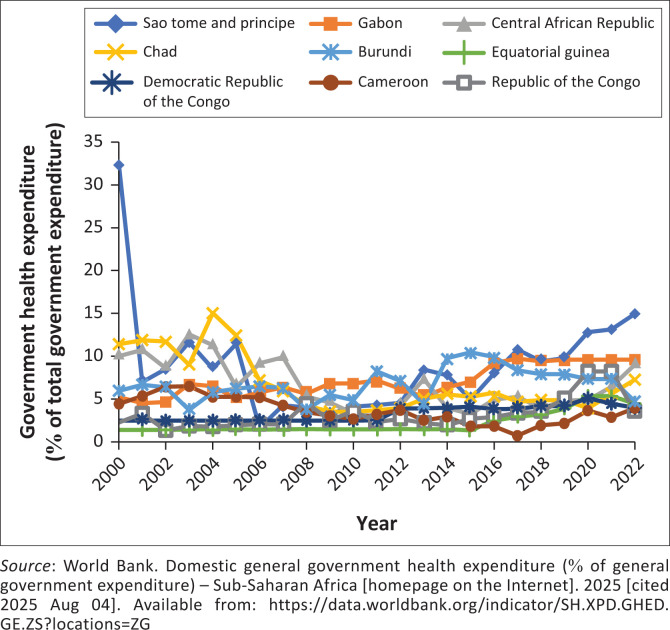
Government health expenditure as a proportion of total government expenditure in Central Africa, 2000-2022.

**FIGURE 6:**
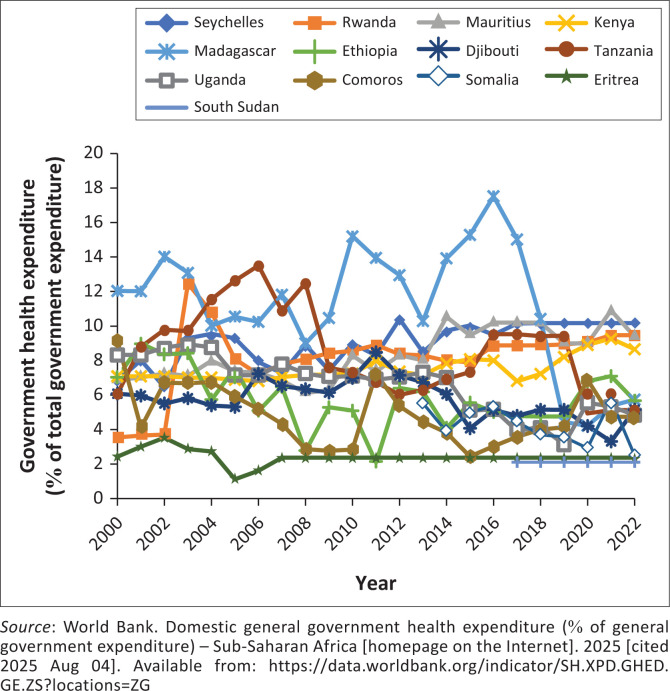
Government health expenditure as a proportion of total government expenditure in Eastern Africa, 2000-2022.

**FIGURE 7:**
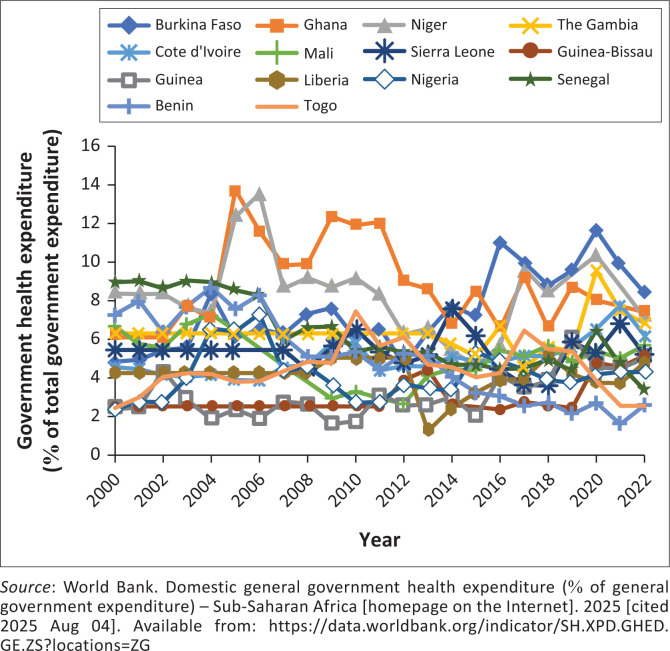
Government health expenditure as a proportion of total government expenditure in Western Africa, 2000-2022.

Even when the argument of competing development priorities is considered, many countries in the continent are not allocating enough to health services. Of the 53 AU Member States analysed, 16 (~30%) allocated less than 5% of their total government expenditure to the health sector in 2022. This finding highlights the extent to which health remains a low budgetary priority in a significant share of AU Member States, irrespective of income level, with important implications for health system services and health outcomes.

## Discussion

As the analysis demonstrates, while economic growth and fiscal capacity are necessary enablers of domestic health financing, the substantial heterogeneity in GHE observed across countries and regions underscores the critical role of political commitment and institutional stability. Scaling domestic investments in healthcare is essential and there is evidence that well-funded health systems enable individuals and communities to prevent ill health, avoid catastrophic health spending, and contribute to economic activity, resulting in high social and economic returns overall.^13^

In this section, we examine these findings in detail, exploring strategies for mobilising domestic resources for health with a focus on country-level political and economic constraints and their implications for contemporary health financing in the continent given reductions in ODA.

### Expanding fiscal space for health: Constraints and opportunities

In line with Heller’s framework, African policymakers seeking to expand fiscal space for health must understand the trade-offs associated with macroeconomic growth, domestic revenue mobilization, efficiency gains, and expenditure reprioritisation.

Firstly, macroeconomic growth remains a limited and uncertain pathway for expanding fiscal space. Despite modest economic growth projections for sub-Saharan Africa, economic expansion has been insufficient to offset raising debt servicing costs, inflationary pressures and post-pandemic fiscal consolidation.^14^ In part, this explains why several AU Member States experienced stagnation or declines in GHE as a proportion of total government expenditure following the COVID-19 pandemic even when nominal budgets increased.

Secondly, domestic revenue mobilisation through taxation offers a more controllable but politically sensitive approach. Country experiences from Rwanda, Kenya, and Ghana show that tax-based financing can expand health coverage; however, the regressive nature of consumption taxes and excise duties poses equity risks.^15,16,17^ This reinforces the idea that revenue expansion must be accompanied by compensatory social protections and progressive tax design to avoid undermining health equity particularly in contexts where informal economies are dominant.

Thirdly, efficiency gains are one of the most politically feasible yet underutilised sources of fiscal space. Investments in public financial management, performance-based budgeting and digital health transformation offer opportunities to ‘do more with less’, particularly where outright spending increases are fiscally constrained.

Fourthly, expenditure reprioritisation appears to be the most challenging path for policymakers. While the Abuja Declaration established a clear benchmark, the fact that only one AU Member State met the 15% target in 2022 illustrates the difficulty associated with shifting resources towards health in the midst of competing priorities. Nigeria’s fuel subsidy reform exemplifies both the potential and the pitfall of reprioritisation. Although fiscally rational, the reform provoked widespread protests, emphasising the importance of transparency, communication and trust in designing and implementing policy reforms.^18^

Ultimately, expanding fiscal capacity for health in Africa requires a tailored, multipronged approach that considers economic conditions, political realities and institutional capabilities. While each strategy presents risks, a balanced combination of tax policies, efficiency gains, enhanced domestic resource mobilisation and efficient prioritisation of programmes can strengthen healthcare systems while mitigating unintended consequences. Strong governance, adaptability and equitable resource allocation are essential to achieving universal health coverage and improving health outcomes across the continent.

### Understanding the political economy of Africa’s health financing systems through ideas, interests and institutions

Using the ‘3Is’ framework, the authors examined how ideas, interests, and institutions shape the allocation of domestic health sector resources in Africa.^11^ Countries such as Rwanda and Mozambique with significant increases in their domestic health budget allocation since the Abuja Declaration appear to have internalised the *idea* of health spending as a foundational investment, allocating relative high shares of their budgets despite limited fiscal capacity. On interests, the persistence of low health allocations in many countries reflects the relative weakness of health constituencies in national budget negotiations, particularly where health benefits are diffuse and long-term, while the costs of reform are immediate and concentrated. This is coupled with the fact that weak *institutions* amplify short-termism and make health budgets vulnerable during fiscal shocks, as observed in post-pandemic expenditure declines in many countries.

### Contemporary regional approaches and implications for Africa’s health financing

The Abuja Declaration represents a bold and ambitious call for AU Member States to strengthen domestic resource mobilisation for health. However, achieving its financing target is unlikely in the absence of clear, credible, and sustained national strategies to scale-up domestic budgetary allocations to the health sector.

In July 2024, health ministers from nine African countries convened in Abidjan for a high-level dialogue on sustainable immunisation financing. Structured as a forum for sharing lessons and addressing common regional health threats, the dialogue stressed the importance of regional solidarity in future-proofing population health and strengthening health security.^19^

However, similar to the Abuja Declaration, the forum did not articulate concrete implementation plans or clearly defined timelines for delivering agreed commitments. In the absence of a publicly available accountability framework, sustaining momentum around these public health priorities will be challenging in an increasingly fluid and constrained global health financing environment. Embedding a formal governance and reporting framework within existing WHO AFRO-led health ministers’ platforms could enable participating countries to regularly report on progress towards agreed targets. Such regional coordination mechanisms would support systematic monitoring, enhance accountability and facilitate course correction where necessary. In the context of declining ODA, ensuring the efficient allocation and use of existing domestic resources becomes even more critical.

Furthermore, the Africa Centres for Disease Control and Prevention (Africa CDC) has outlined recommendations to mitigate the constraints posed by declining ODA.^20^ Through the Lusaka Agenda, a consultative process encompassing diverse stakeholders in the continent’s health ecosystem, the agency, Africa’s flagship public health institution highlights several key approaches. These include mobilising greater domestic resources by updating national strategic plans to reflect evolving priorities and shifts in the global health financing landscape, while ensuring that external support is aligned with these plans. Additional measures include scaling-up innovative financing mechanisms, leveraging blended finance from development finance institutions, and strengthening regulatory ecosystems to attract more private investment into the health sector.

## Conclusion

By examining trends in domestic health financing across five regional blocs in the AU, this article identifies countries and regions that have made substantial progress, as well as those that have experienced stagnation or decline. Several methodological limitations should, however, be acknowledged. These include the largely descriptive nature of the analysis, gaps in country-level data for certain years, and the non-causal design of the study, all of which limit the generalisability and interpretability of the findings.

Overall, the evidence suggests that expanding fiscal space for health in Africa requires more than aspirational pledges and high-level declarations. Domestic resource mobilisation has become both critical and urgent, particularly in the context of recent reductions in ODA from high-income countries. Strengthening health system financing in low- and middle-income countries will require sustained political commitment, institutional capacity building, expanding innovative financing models and a deliberate reframing of health as a strategic investment rather than a discretionary social expenditure. The interaction between domestic political economy dynamics and a contracting global aid environment further heightens the urgency for structural reform in health financing across the continent.

A pragmatic path forward lies in combining incremental domestic resource mobilisation, efficiency improvements and selective reprioritisation, while strengthening institutions that anchor health financing over the political cycle. In an era of declining external support, countries that align ideas, interests and institutions around health financing will be best positioned to achieve resilience, self-reliance and progress towards universal health coverage.
